# SLC30A1, SLC30A5, and SLC30A9 transporters play crucial role in ligand-independent activation of ESR1 signalling in breast cancer cells via modulation of AKT activity by zinc

**DOI:** 10.1093/mtomcs/mfag011

**Published:** 2026-03-06

**Authors:** Szymon Lekki-Porębski, Michał Rakowski, Agnieszka Grzelak

**Affiliations:** Centre for Digital Biology and Biomedical Science - Biobank Lodz, Faculty of Biology and Environmental Protection, University of Lodz, Lodz 90-236, Poland; The Bio-Med-Chem Doctoral School of the University of Lodz and Lodz Institutes of the Polish Academy of Sciences, University of Lodz, 90-237 Lodz, Poland; Centre for Digital Biology and Biomedical Science - Biobank Lodz, Faculty of Biology and Environmental Protection, University of Lodz, Lodz 90-236, Poland; Centre for Digital Biology and Biomedical Science - Biobank Lodz, Faculty of Biology and Environmental Protection, University of Lodz, Lodz 90-236, Poland

## Abstract

Zinc is essential for cellular homeostasis and acts both as a structural element and a secondary messenger in intracellular signalling. While the role of SLC39 (former ZIP) family transporters in breast cancer biology is intensively studied, the signalling function of SLC30 family transporters (former ZnT) remains insufficiently explored. This study investigates the involvement of SLC30 transporters in oestrogen receptor-positive (ER+) breast cancer. Bioinformatic and experimental analyses revealed that SLC30 transporters, particularly SLC30A1, SLC30A5, and SLC30A9, regulate the PTP/AKT/ESR1 pathway, contributing to hormone-independent ESR1 activation. Zn-dependent inhibition of PTP phosphatases modulates kinase signalling, promoting proliferation. Notably, high SLC30 expression correlates with improved survival, but serves as a negative prognostic marker under tamoxifen treatment. Here, we evidence that ESR1 directly represses SLC30 transcription and that zinc transporters form a regulatory feedback loop sustaining ER+ breast tumour progression. These findings position SLC30 transporters as active participants in signalling cascades, offering novel targets for therapeutic intervention in ER+ breast cancer.

## Graphical Abstract

**Graphical Abstract:**
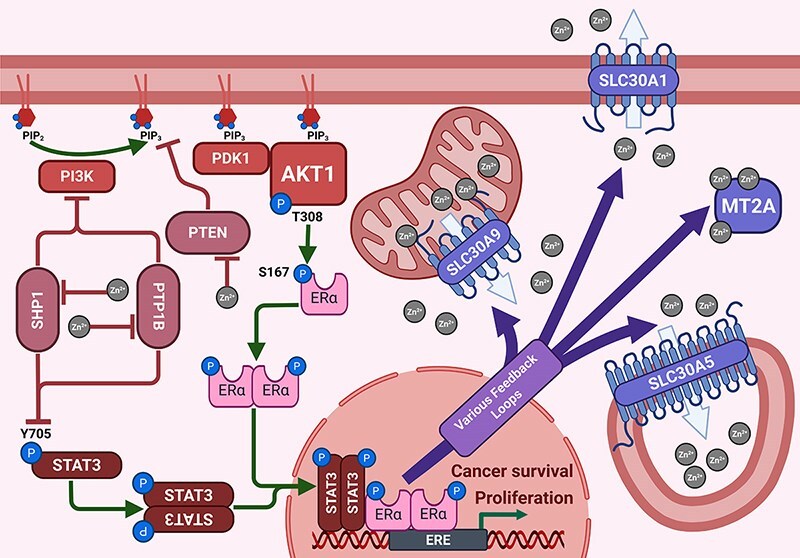

## Introduction

Zinc is a pivotal micronutrient, a key player in the maintenance of human body homeostasis. More than 10% of human proteins permanently or transiently bind the Zn^2+^ ion in their tertiary structure. To assure physiological homeostasis for zinc, humans developed two major families of transmembrane zinc transporters: SLC30 and SLC39 [1]. The SLC39 transporters (SLC39A1-SLC39A14; also known as ZIP) are responsible for the transport of zinc across membranes into the cytoplasmic space. In contrast, SLC30 transporters (SLC30A1-SLC30A10, former ZnT) pump zinc out of the cytoplasmic space (into organelles and into the extracellular space, with exception to SLC30A9). While those two are basically importers and exporters, the metallothionein family (MT1–MT4) is primary responsible for storage functions in the cell. Moreover, metallothioneins play role in zinc redistribution and free radicals scavenging. Additionally, MTF-1 promotes transcription of proteins maintaining zinc homeostasis in the cell. The expression, activity and specific localization of individual zinc transporters results in Zn^2+^ concentrations in blood and organelles ~10^−5^ mol/dm^3^, whereas Zn^2+^ concentrations in the cytoplasm generally do not exceed 10^−10^ mol/dm^3^ [2].

In addition to zinc’s structural role in proteins, it also serves as a secondary signal messenger. Changes in intracellular zinc concentration regulate the activity of phosphorylation-dependent signalling pathways, including: ERK1/2, STAT1/3, PTPs, PKA, or NF-κB [3]. Consequently, the activity of zinc transporters exerts an indirect regulatory influence on the pathways implicated in tumorigenesis (via zinc as secondary messenger), suggesting that it may play a crucial role in the development of breast cancer. Many results supporting this hypothesis have been published so far. For instance, high dietary zinc levels are correlated with better survival of breast cancer patients [4]. Possibly because mammary gland tumour tissue has an increased ability to accumulate zinc when compared to healthy tissues [5]. Among both families of zinc transporters, role of SLC39 in breast cancer is better established. SLC39 transporters have been shown to regulate the cell cycle, proliferation, and metastasis of breast cancer. For example, activity of SLC39A6/SLC39A10 is crucial for progression of breast cancer cell through mitosis [6]. SLC39A7 was associated with proliferation of breast cancer [7]. SLC39A6 was proposed as a marker of low metastatic potential in breast cancer [8].

Despite the advances in understanding of the signalling role of zinc, the function of SLC30 transporters in the regulation of intracellular signalling is still poorly understood. This study aimed to analyse the role of SLC30 transporters in the regulation of oestrogen-dependent signalling. *In silico* analysis indicated that patients with ER+ breast cancer often have an overexpression of SLC30 transporters, suggesting that SLC30 transporters could serve as a prognostic indicator. Conversely, in data derived from patients treated with tamoxifen, such overexpression functions as a negative prognostic indicator. Here, we present preliminary studies suggesting that zinc may modulate proliferation of ER+ cells via PTP/PI3K/AKT/ESR1 axis. Additionally, we show that SLC30 transporters (i.e. SLC30A1, SLC30A5, and SLC30A9) can be regulated through oestrogen-dependent signalling.

## Materials and methods

### Cell line and treatment

MCF-7 epithelial breast cancer cell line (ATCC^®^ HTB-22, ER+, PR+, GPER1+, HER2-) was used for this study. MCF-7 cells were cultured according to ATCC recommendation using full-growth medium (MEM low glucose with HEPES and Phenol Red, ThermoFisher Scientific) supplemented with 5 nmol/dm^3^ 17-β-estradiol, NEAA, L-glutamine and 10% (v/v) FBS [9]. Experiments were performed using phenol red-free, full-growth medium supplemented with 10% (v/v) carbon stripped FBS. Aqueous solution ZnSO_4_ × 7 H_2_O was used as a zinc donor in all experiments (Cat. No 265 750 119, POCh S.A., Gliwice, Polska). Cells were incubated with either a zinc donor or with a mixture of selected factors at following final concentrations: ZnSO_4_ (20 μmol/dm^3^), EDTA (50 μmol/dm^3^), tamoxifen (400 nmol/dm^3^), 17-β-estradiol (E2, 10 nmol/dm^3^), capivasertib (200 nmol/dm^3^), chloramphenicol (5 μmol/dm^3^), ulixertinib (100 nmol/dm^3^), LPS from *E.coli* O127: B8 (150 ng/cm^3^), nilotinib (300 nmol/dm^3^). Concentration of following factors was chosen based on viability curves as highest non-toxic in our model: Tamoxifen, Capivasertib, Chloramphenicol, Ulixertinib, Nilotinib, LPS. Concentrations of EDTA and E2 were chosen based on literature [10,11]. Concentration of zinc donor was chosen as highest acceptable blood zinc concentration in healthy human [12]. Viability curves for each treatment are available in Supplementary Fig. S1.

### Proliferation assay

The measurement of MCF-7 cells proliferation was performed using a resazurin assay. 2 × 10^3^ cells in 0.1 cm^3^ of full-growth media were seeded in 96-well plates with flat, black bottoms for fluorescence measurement (Nunc, Thermo Fisher Scientific). Twenty-four hours after seeding, selected compounds were added in 0.1 cm^3^ of culture medium to achieve final concentration. After the incubation period, culture medium was removed and resazurin solution (in 1X concentrated HBSS) was added to the final resazurin concentration of 15 μg/cm^3^. Fluorescence signal was measured every 20 min (120 min total) at Ex/Em wavelengths 571/585 nm. The results were calculated as a slope value over time and normalized to control values. Doubling time of cells was calculated based on Malthusian growth model. Statistical analysis of results was performed using multiple *t*-student test at α = 0.05.

### RT-qPCR assay

Total RNA was isolated using a commercially available kit (Syngen Blood/Cell RNA Mini Kit, Syngen Biotech) according to the manufacturer’s instructions. The quality and quantity of the RNA were assessed using a Nanodrop spectrophotometer (ThermoFisher Scientific). A reverse transcription assay was conducted using the Maxima First Strand cDNA Synthesis Kit according to the manufacturer’s instructions (ThermoFisher Scientific). The RT-qPCR assay was performed using SsoAdvanced Universal SYBR Green Supermix (BioRad). Primer sequences can be found on Supplementary Fig. S2. The RT-qPCR reaction (in total sample volume of 10 mm^3^) was performed as follows: 2 min at 95°C (hot start), 40 cycles consisting of 5 s at 95°C (denaturation) and 30 s at 60°C (annealing, polymerization, and fluorescence measurement). A melting curve was generated for each sample in a temperature range between 65°C and 95°C (0.5°C step). Representative melt curves are presented in Supplementary Fig. S3. The results were expressed as Ct from an automatic regression calculation and standardized using housekeeping genes (*GAPDH* and *RPL13A*). Results were analysed using non-parametric ANOVA followed by Welch correction as post-hoc test at α = 0.05.

### Western blot assay

5 × 10^3^ cells in 3 cm^3^ of full-growth media were seeded in 6-well plates. Twenty-four hours after seeding, zinc and/or capivasertib was added. After incubation, cells were washed once with PBS buffer and lysed using M-PER solution with Halt protease and phosphatase inhibitor cocktail (ThermoFisher Scientific). Lysate was collected and centrifuged for 10 min at 14 000 × *g*. Protein concentration was determined using Pierce BCA assay. Samples were mixed with loading buffer and incubated for 5 min at 95°C. Eight micrograms of protein sample was loaded per well of 10% polyacrylamide gel. Electrophoresis was performed in SDS-PAGE buffer at 130 V for ~1.5 h. The proteins were transferred from the polyacrylamide gel to PVDF membranes using the Trans-Blot^®^ Turbo™ Transfer System (Bio-Rad). Membranes were incubated overnight in blocking buffer (1% IgG-free BSA in TBST). After blocking, membranes were incubated overnight with following primary antibodies: rabbit anti-p-AKT1 (T308) (AF0832, Affinity Biosciences, dilution 1:2000), rabbit anti-AKT1 (AF0836, Affinity Biosciences, dilution 1:2000), and anti-β-ACT (AC026, ABclonal, dilution 1:160 000). Next, membranes were washed five times in TBST solution for 5 min and incubated for 1 h with anti-rabbit goat secondary antibodies (Vector Labs, dilution 1:50 000). Membranes were washed five times and incubated for 5 min in the imaging solution (Thermo Fisher Scientific). Images were taken using an UVITEC Cambridge Alliance HD4 Mini chemiluminescence analysis device (UVITEC, Cambridge, UK). Sample analysis was performed using UVITEC Alliance software. Results for total AKT1 level are presented in Supplementary Fig. S6. Original chemiluminescence and light photographs of membranes are presented in Supplementary Fig. S7.

### *In silico* analysis

*In silico* study was performed by reanalysis of datasets derived from breast cancer patients using publicly available sources collected by author mentioned below. Genes from *SLC30* family expressed in breast cancer and *MT2A* genes were reanalysed for differential expression and survival analysis in Either All Patients or tamoxifen-only treatment only groups. Only data from ER+ tumors was used for the analysis. Survival analysis and Kaplan–Meier plots were prepared using the Kaplan–Meier Plotter software [13]. A Recurrence-free Survival (RFS) data with percentile cutoff method for high and low expression group selection was used. Log-rank test has been utilized to calculate the statistical difference. Differential expression of selected genes was prepared using gene expression profiling interactive analysis (GEPIA2) software [14]. Following parameters setup was used for analysis: “TCGA tumours vs TCGA normal + GTEx normal”; |log_2_FC| Cutoff = 0.5 and *P*-value threshold = 0.05 were used for analysis. Due to low expression in breast cancer group (TPM ≤ 0.5), RNA-Seq and Kaplan–Meyer plots of following genes have not been included for this study: *SLC30A2, SLC30A3, SLC30A8*, and *SLC30A10* [15]. RNA-Seq box-plots and Kaplan–Meyer plots for these genes are presented in Supplementary Fig. S4. Correlation between expression of *SLC30A1, SLC30A5*, or *SLC30A9* and a transcript signature of specific pathway activation (i.e. AKT1, PRKCD, and RAF1) was prepared using GEPIA2 software. List of genes used as the pathway signature (including original publication) are presented in Supplementary Fig. S5. Regulation of *SLC30A1, SLC30A5*, or *SLC30A9* was assessed using Toolkit for Cistrome Data Browser (Cistrome DB toolkit) [16]. The half-decay distance to transcription start site was set to 100 kb (to include both TF- and enhancer-like regulation) and results were selected for samples derived from either breast cancer tissue or breast-derived cell line.

## Results

### *SLC30* genes are overexpressed in ER+ breast cancer and affect survival rate of patients

**Figure 1:**
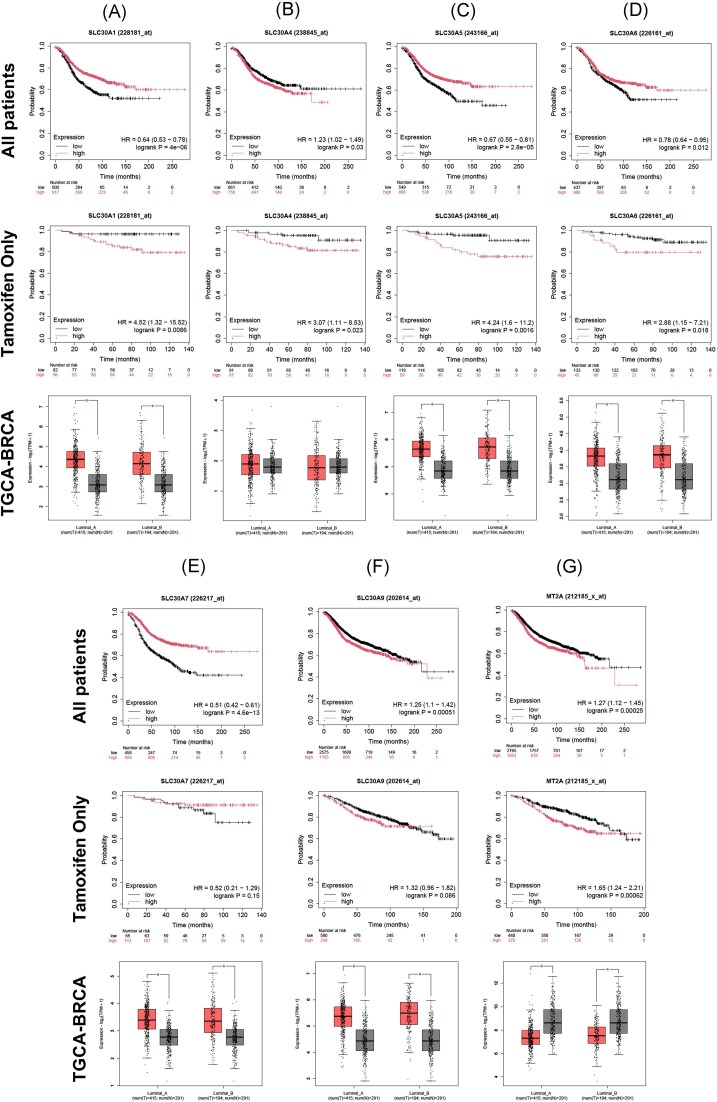
Correlation between expression of selected genes and survivability of ER+ breast cancer patients: *SLC30A1* (A), *SLC30A4* (B), *SLC30A5* (C), *SLC30A6* (D), *SLC30A7* (E), *SLC30A9* (F), and *MT2A* (G). Kaplan–Meyer plots represent data of RFS in All ER+ breast cancer patients’ group (upper graph) and tamoxifen only treated subgroup (middle graph). Analysis of RNA-Seq data from TGCA-BRCA dataset in different types of ER+ breast cancer (lower graph). Comparison of patients (red boxplot) was performed to healthy control group (grey boxplot). Patients numbers in time for Kaplan–Meyer Plots are presented below each graph. Number of samples for each breast cancer subtype of TGCA-BRCA plots are presented below each graph (num(T)—number of breast cancer patients, num(N)—number of healthy donors). HR—Hazard Risk ratio.

Reanalysis of RNA-Seq data of *SLC30* family of genes (Fig. 1 TGCA-BRCA) revealed that ER+ breast cancers overexpress proteins from this family when compared to tissues from healthy donors with one exception—*SLC30A4*. Although literature confirms that *SLC30A4* is expressed at protein level in breast [15], its’ level is the smallest among *SLC30* expressed in breast tissue. Furthermore, analysis of *MT2A* transcription in ER+ tumours revealed a statistically significant decrease of mRNA level compared to the control group. Analysis of RFS data revealed that the expression of *SLC30A1, SCL30A5, SLC30A6*, and *SLC30A7* genes is correlated with a favourable prognosis, while the expression of *SLC30A4, SLC30A9*, and *MT2A* has been shown to be a predictor of poor prognosis in terms of patient survival rates (all patients group). A significantly different trend was observed in the RFS analysis of the subgroup of patients treated with tamoxifen when compared to all patients. For patients treated with tamoxifen the expression of *SLC30A1, SLC30A5*, and *SLC30A6* was identified as a predictor of poor prognosis while *SLC30A7* showed no association. Meanwhile higher expression of *SLC30A4, SLC30A9*, and *MT2A* correlated with higher hazard risk ratio in the group of patients treated with tamoxifen.

**Figure 2:**
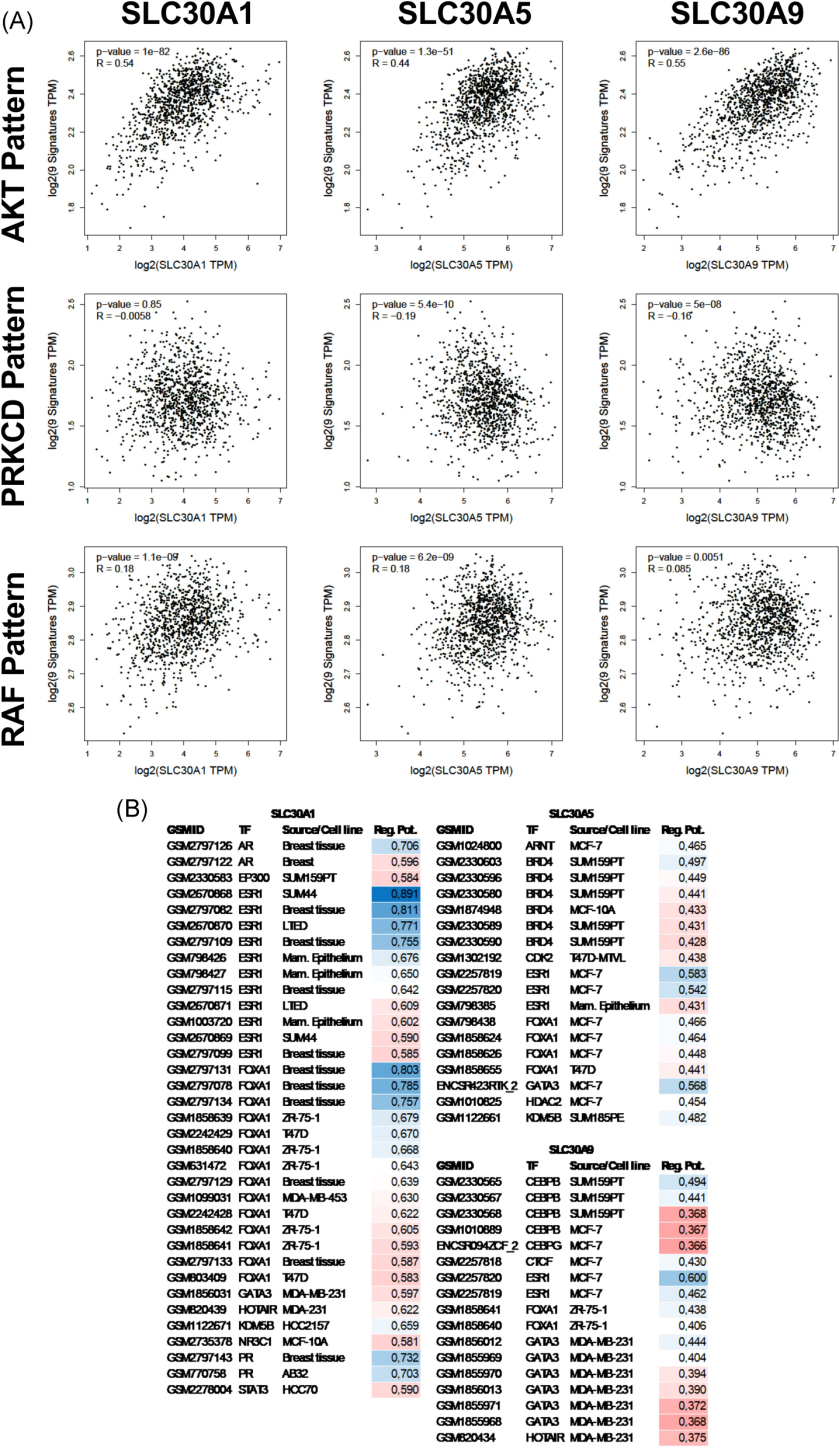
Expression of selected genes in MCF-7 cells treated with either zinc or a mixture of zinc with other compounds. Results were normalized to mean control group value. (A) Heatmap of gene transcription after incubation. (B–F) Transcription of selected genes after 24-h incubation. Results are presented as median values with interquartile range and total range. *P*-values of post-hoc test for Non-parametric ANOVA (between all samples) are presented below each graph of specific gene (total *n* at least 7).

The expression of zinc-induced marker of ESR1 activity and tested SLC30 genes was modulated by the addition of AKT1 and ESR1 inhibitors. Based on results from Fig. 1, we choose six *SLC30* transporters to verify effect of zinc or selected inhibitors on gene transcription (Fig. 2A). Moreover, two additional genes were chosen: *MT2A* and *TFF1. MT2A* was chosen as a marker of the bioavailability of zinc ions (Fig. 2C). *TFF1* is a consensus gene, commonly used as marker of an ESR1 activity [17]. Statistically significant results for tested *SLC30* genes were observed in case of three transporters: *SLC30A1, SLC30A5*, and *SLC30A9* (Fig. 2A). Further research demonstrated that cells supplemented with Zn^2+^ had higher expression of *TFF1, MT2A*, and *SLC30A1* (Fig. 2B–D) and lower gene expression of *SLC30A5* (Fig. 2E). Upregulation of *TFF1* expression was abolished by tamoxifen and capivasertib. Treatment with capivasertib led to upregulation of *MT2A* expression, while no differences in expression of *MT2A* were found between samples treated with capivasertib and samples treated with capivasertib and Zn^2+^ (Fig. 2C). Furthermore, supplementation of cells with Zn^2+^, tamoxifen or chloramphenicol led to an upregulation of *SCL30A1* expression (Fig. 2D). Capivasertib alone decreased the expression of *SLC30A1* mRNA level, which was reversed back to control level in samples from cells supplemented with Zn^2+^. Incubation with Zn^2+^ decreased transcription of *SLC30A5* (Fig. 2E), whereas incubation with tamoxifen increased its expression (regardless of Zn^2+^). Chloramphenicol increased *SLC30A5* mRNA levels, which was abolished by Zn^2+^ supplementation. Furthermore, the results demonstrate that tamoxifen, capivasertib and chloramphenicol increased SLC30A9 transcript levels regardless of Zn^2+^ supplementation (Fig. 2F).

### Selected *SLC30* genes correlates with activity of AKT and are regulated by ESR1

**Figure 3:**
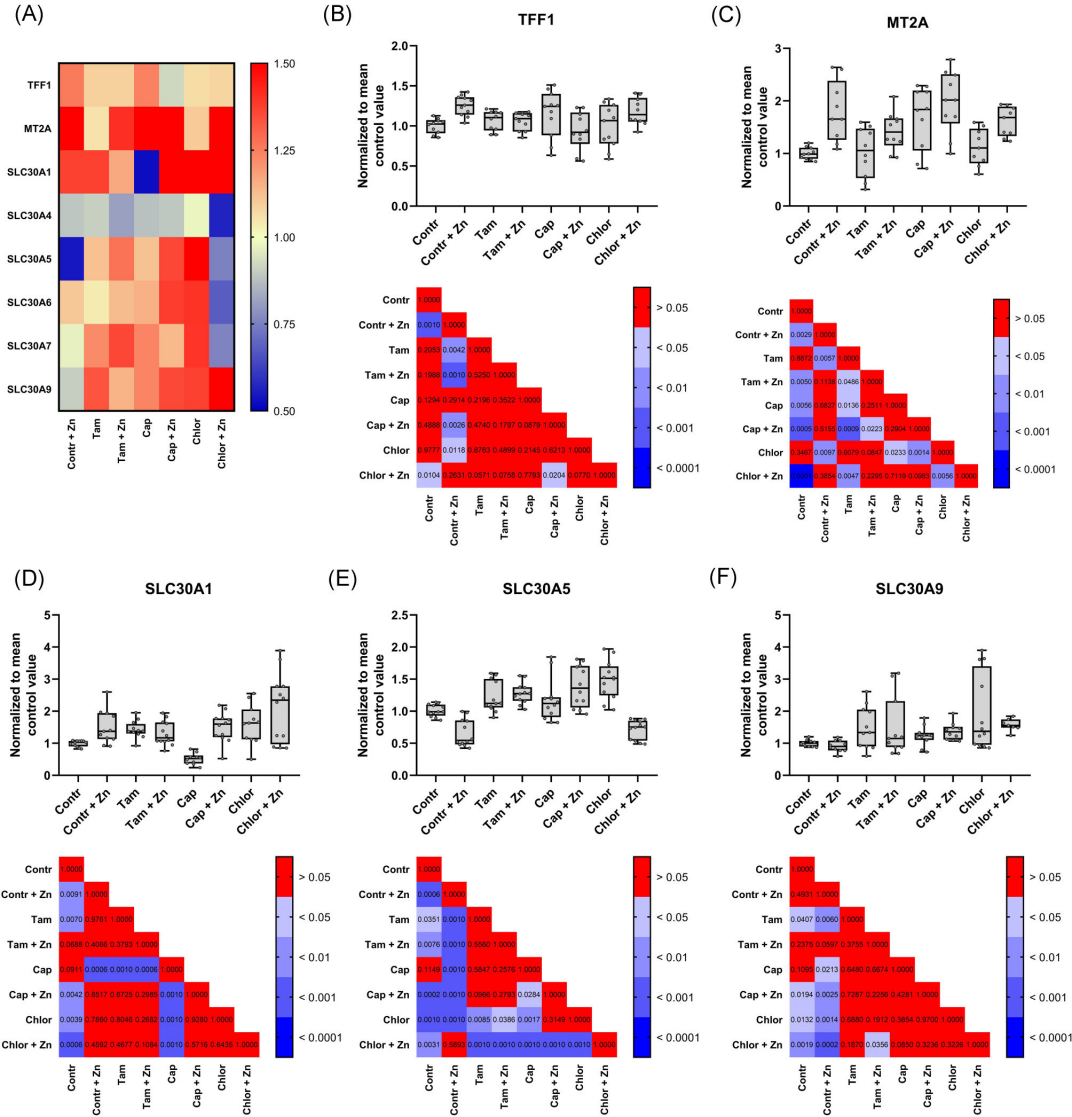
Correlation between expression of selected *SLC30* genes and transcriptional signature of activation of selected signalling pathway (A). Results were presented as *P*-value and Spearman R parameter. Correlation analysis was performed from dataset of breast cancer samples using GEPIA2 software (dataset *n* = 1098). List of transcription factors regulating selected *SLC30* genes in breast derived samples (B). Transcription factors were ordered according to alphabetical order and descending regulatory potential. Colour of regulatory potential value was used for readability of figure B. Analysis of regulatory potential was performed using Toolkit for Cistrome Data Browser (DBtoolkit) (dataset *n* = 47 000). GSMID—GEO accession number of sample; TF—Transcription Factor; Reg. Pot.—regulatory potential.

Current state of knowledge confirms that zinc affect directly activity of several signalling pathways. Expression of *SLC30A1, SCL30A5*, or *SLC30A9* was correlated with transcriptional signature of specific pathway activation (Fig. 3A). Expression of all tested transporters correlated with AKT1 activation pattern (*R* > 0.44). In case of PKCδ activation pattern (PRKCD) statistically significant results with negative *R*-value were observed for *SCL30A5* or *SLC30A9* genes. For RAF1 activation pattern, results with positive *R*-value were observed far all selected SLC30 genes. Despite this, absolute *R*-value for RAF1 and PRKCD were <0.19. Moreover, graphical analysis did not show any linear-like pattern in RAF1 or PRKCD activation pattern in contrast to AKT1 activation pattern. Analysis of transcription factors Regulatory Potential (RP) using Toolkit for Cistrome Data Browser (DBtoolkit) confirmed that all three *SLC30* genes were *in silico* regulated by ESR1 (Fig. 3B). Moreover, expression of researched SLC30 genes was regulated by FOXA1—mostly in samples derived from ESR1+ cells. Only *SLC30A5* was regulated by BRD4. Only *SLC30A9* was regulated by CEBPB. Additionally, *SLC30A1* was regulated by other steroid hormone receptors (i.e. AR and PR).

### Zinc promotes proliferation of MCF-7 cells via activation of AKT1

**Figure 4:**
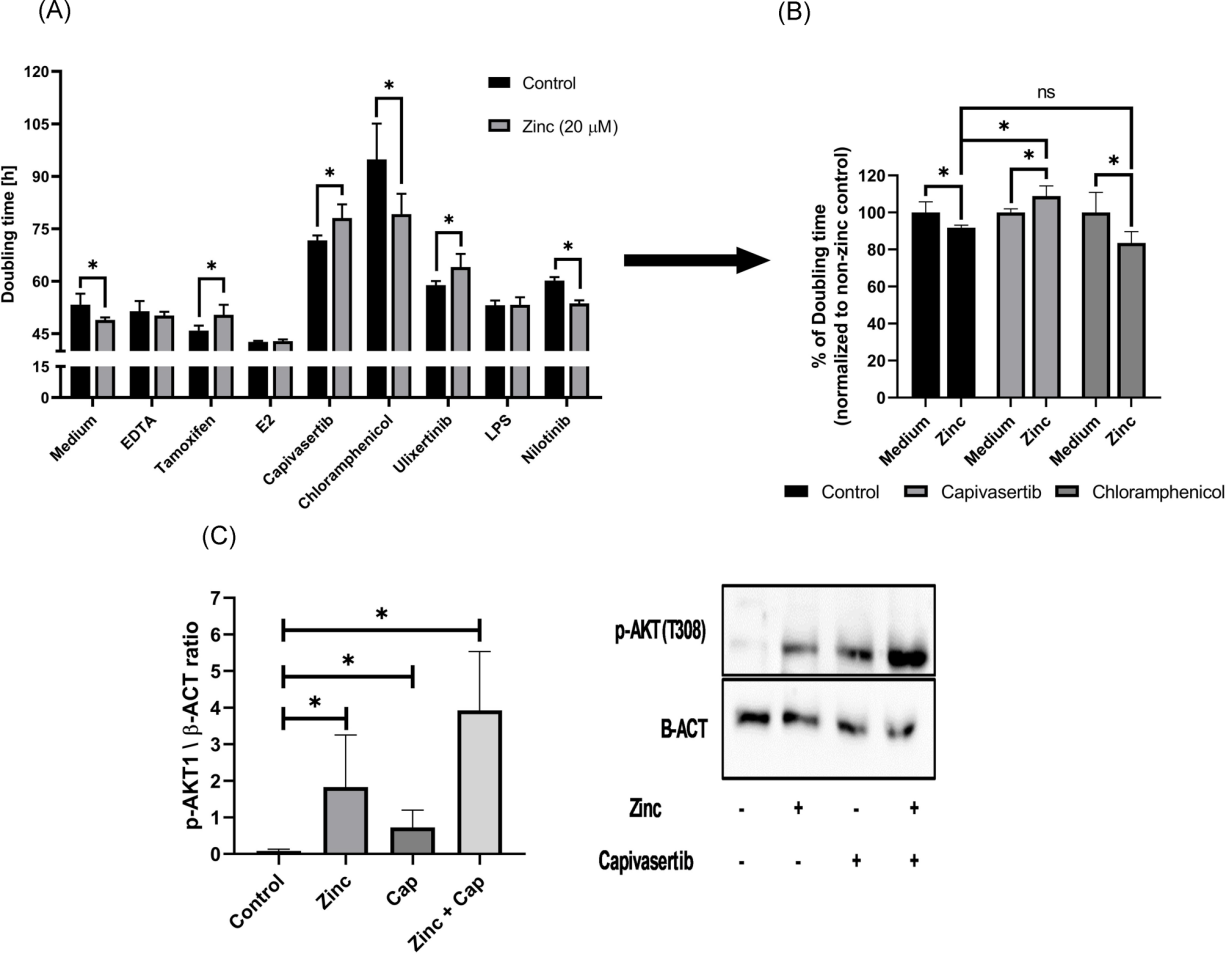
Doubling time of cells after incubation with either zinc or mixture with selected factors (A). Results were analysed using paired multiple *T*-test [total *n* = 12]. Doubling time normalized in each group to mean of zinc-free control samples (B). Results were analysed using parametric ANOVA [*n* = 12]. Western blot of p-AKT1 (T308) normalized to β-actin (C). Results were analysed using non-parametric ANOVA [*n* = 3]. *—*P* < 0.05.

Zn^2+^ ions boosted doubling time of MCF-7 cells (Fig. 4A), which was abolished by EDTA. Analysis of MCF-7 cell proliferation (Fig. 4A and B) demonstrates that tamoxifen abrogates the proliferative effect of Zn^2+^ ions, while E2 accelerates the effect regardless of the presence of zinc. Interestingly, when tamoxifen, capivasertib or ulixertinib were added to the cells alongside Zn^2+^ treatment, the observed induction of proliferation was reversed, resulting in longer doubling time of cells when compared to control sample. Chloramphenicol and nilotinib did not exhibit any significant impact on the proliferative effect of Zn^2+^. Normalization of results revealed that capivasertib inhibited Zn-induced proliferation boost while chloramphenicol did not (Fig. 4B). Treatment of cells with capivasertib (an AKT inhibitor) and ulixertinib (an ERK1/2 inhibitor) reversed the proliferative effect of Zn^2+^. Treatment of MCF-7 cells with LPS abolished zinc-dependent proliferation boost. AKT1 activity was analysed using phospho-Blot against p-AKT1 at T308 (marker of AKT1 activation) [18]. Results showed an increase in level of p-AKT1 after treatment with Zn^2+^ or capivasertib (Fig. 4C). Co-treatment with both Zn^2+^ and capivasertib led to cumulative effect. No changes were observed in total AKT1 level (Supplementary Fig. S7).

## Discussion

This study elucidates the interplay between ESR1-dependent signalling and SLC30 family of zinc transporters. While it is well established that zinc plays a pivotal role in the homeostasis and pathology of the mammary gland, the detailed mechanistic explanation of this phenomenon remains incompletely understood. Our data confirm a significant link between breast cancer and zinc homeostasis, evidenced by the overexpression of SLC30 transporters in ER+ breast cancer cells compared to patients’ data (Fig. 1).

Here, we suggest an inverse relationship between SLC30 expression and MT2A levels. It seems, that SLC30 proteins, through active reduction of the level of free zinc ions, downregulate the activity of MTF-1 leading to a decrease of its binding potential to the MRE sequence. This, in turn, results in reduced expression of the MT2A gene, which protein product is crucial for regulation of intracellular zinc level. This presents a paradox, as mammary tumor tissues typically possess an increased capacity to accumulate zinc compared to healthy tissue [19]. We propose that this effect is driven by the SLC30 transporters which actively import zinc into organelles, thereby reducing the pool of free cytoplasmic zinc ions [20]. This, in turn, diminishes the capacity for the induction of MT2A expression. Furthermore, lower intracellular zinc marker levels corelate with increased patients’ survival rate (Fig. 1G), suggesting that zinc may function as an inhibitor of signalling proteins that are responsible for disease progression in ER+ tumours [3].

Analysis of RFS did not prove a direct interplay between Zn^2+^ and ESR1 signalling in ER+ breast cancer patients, but additional analysis of the tamoxifen-treated subpopulation supports this hypothesis. Our results reinforce the hypothesis that Zn^2+^ stimulates the ESR1 pathway in the absence of a hormonal ligand. To date, multiple different routes for activation of the ESR1 have been described. Zinc seemed to activate the ESR1 during the absence of E2, leading to phosphorylation of the ESR1 catalysed by, most likely, AKT1 [21] (Fig. 4C). This kinase is regulated by phosphatases from the PTP family, such as PTEN, SHP1, and PTP1B. Interestingly, zinc in physiological concentration inhibits all of these phosphatases [22], with such effect observed in our proliferation assays utilizing inhibitors and/or activators of selected intracellular pathways (Fig. 4A and B). It is worth noting that AKT and ERK1/2 pathways are both regulated by phosphatases from the PTP family [23]. The lack of relative acceleration of proliferation (Fig. 4A) by both nilotinib (indirect activator of ERK1/2) and chloramphenicol (indirect AKT activator) suggests that inhibition of phosphatases, rather than the direct activation of AKT1 was the critical factor in the regulation of pathways by Zn^2+^. The hypothesis describing phosphatases as a direct target for zinc was supported by analysis of transcriptional signatures of pathway activation (Fig. 3A). In addition to phosphatases, zinc directly regulates activity of kinases such as RAF1 or PRKCD by direct binding to them [24,25]. Only the AKT1 pathway signature seemed to be correlated with the level of selected SLC30 transporters. Thus, it can be concluded that zinc-sensitive factors regulating AKT1 (i.e. phosphatases) were zinc’s primary point of action on the AKT1/ESR1 axis.

In addition to AKT and ERK proteins, PTP phosphatases inhibit STAT3-dependent signalling [26]. LPS is known for its modulation of STAT3 signalling via the TLR pathway. Abolition of the zinc-dependent proliferation boost by LPS (Fig. 4A) suggested that STAT3 may be one of the factors mediating Zn^2+^ effects on the AKT1/ESR1 axis [27]. One of the potential targets that may be responsible for such effects is the mitochondrial zinc exporter (i.e. SLC30A9). In the absence of a zinc salt, the flux of Zn^2+^ ions from mitochondria to cytoplasm may result in the activation of STAT3, thereby restoring homeostasis within the mitochondria, which ultimately reduces oxidative stress by decreasing ROS and increasing ATP production [28]. This was confirmed by observed upregulation of *SLC30A9* transcription in cells treated with chloramphenicol (Fig. 2F). Chloramphenicol disrupts oxidative phosphorylation in mitochondria. MCF-7 cells responded to this by decreasing the level of zinc through the induction of SLC30A9 level [28,29]. Additionally, STAT3 has been shown to interact directly with ESR1, forming a transcriptional complex that is responsible for breast cancer proliferation and once again linking the STAT/JAK pathway with oestrogen signalling [30]. Moreover, *SLC30A9* is regulated by CEBPB and FOXA1 in ER+ samples (Fig. 3B). These two transcription factors link directly *SLC30A9* with STAT3. Literature confirmed that transcription of the *CEBPB* gene is regulated by STAT3 [31]. CEBPB protein itself is stabilized by direct interaction with STAT3 [32]. FOXA1 together with ESR1 and STAT3 forms a complex in promotor regions of selected genes [30]. Based on these results, we conclude that the *SLC30A9* gene is connector between STAT3 and the zinc/AKT1/ESR1 axis.

We propose the hypothesis, that selected SLC30 transporters were key factors closing the PTP/AKT1/ESR1 axis in positive feedback loop. The proposed mechanism would explain observed changes of SLC30 at the transcription level (Fig. 2D–F) and proliferation of MCF-7 cells (Fig. 4A and B) after incubation with modulators of AKT1 or ESR1. Positive feedback loops in ESR1 signalling are not unusual as classic examples of positive loops have been well described [33]. Analysis of *SLC30* promoters (Fig. 3B) confirmed that ESR1 theoretically together with FOXA1 and CEBPB is capable of regulation transcription of the aforementioned transporters. Both activation and repression cannot be excluded, as ESR1 plays both roles depending on the specific gene and co-regulators associated with ESR1. An example of a co-regulator participating in gene repression by ESR1 is p300 protein [34]. Additionally, p300 protein interacts directly with STAT3, thus its role in transcription of SLC30A1 and SLC30A5 cannot be excluded [35]. Especially as STAT3 interacts with the CEBPB and FOXA1. Mechanistically, improved transport of zinc by SLC30A1 and SLC30A5 proteins out of the cytoplasm would result in an increase of PTPs activity which might subsequently reduce the activity of the AKT/ESR1 axis. The opposite effect would be observed for SLC30A9. The observed decrease of *SLC30A5* proved, that AKT1 via ESR1 functioned as a repressor of *SLC30A5* (Fig. 2E), which is strongly supported by the presence of an ERE element in the regulatory sequence of the gene (Fig. 3B) and it was proven *in vitro* by Kumar et al. [36]. SLC30A1 transports Zn^2+^ from the cytoplasm into the extracellular space to maintain Zn^2+^ ion homeostasis (Fig. 2D), so it is logical that the addition of Zn^2+^ would increase the expression of SLC30A1 [37]. However, stress conditions induced by tamoxifen and chloramphenicol also led to an upregulation of *SLC30A1* transcription. This upregulation might decrease the cytoplasmic level of Zn^2+^ and was driven by inhibition of ESR1, which binds to the promoter of *SLC30A1* (Fig. 3B). Overall, we concluded that upregulation of SLC30A1 and/or SLC30A5 disrupted the positive feedback loop of the zinc/PTP/AKT1/ESR1 axis. A decreased level of cytoplasmic Zn^2+^ led to an increase of PTP activity and downregulation of the AKT1/ESR1 driven proliferation of cells. Moreover tamoxifen-driven stress promoted the transcription of *SLC30A9* (Fig. 2F). Zn^2+^ efflux from mitochondria via SLC30A9 increases the efficiency of the oxidative phosphorylation chain, which is a logical consequence of increased energy demand during stress conditions. Additionally, mitochondria play role as zinc storage organelles [38], thus increased efflux might be expected to reclaim cytoplasmic zinc homeostasis. To summarize, transcriptional regulation of *SLC30A1, SLC30A5*, and *SLC30A9* created a positive feedback loop accelerating PTP/AKT1/ESR1 signalling. The specific mechanism of action might differ, depending on localisation in specific organelles and the physiological function of the selected SLC30 transporter.

In conclusion, our results show that Zn^2+^ ions regulate the activity of the PTP/AKT/ESR1 signalling pathway. Moreover, selected transporters from the SLC30 family led to the creation of a positive feedback loop in. SLC30/Zn/PTP/AKT1/ESR1/SLC30 pathway. This suggests that the SLC30 family may also have a signalling function using the Zn^2+^ ion as a secondary signal transducer. However, further research is needed to understand the exact mechanism of the pathway and to determine which PTP phosphatase plays a key role in this process.

## Supplementary Material

mfag011_Supplemental_File

## Data Availability

The experimental data underlying this article will be shared on reasonable request to the corresponding author. The datasets for bioinformatic analysis were derived from sources in the public domain: GEPIA2 (http://gepia2.cancer-pku.cn/), Kaplan-Meier Plotter (https://kmplot.com/analysis/), Cistrome DB Toolkit (http://dbtoolkit.cistrome.org/).
