# Supplementary material for: SLC30A1, SLC30A5, and SLC30A9 transporters play crucial role in ligand-independent activation of ESR1 signalling in breast cancer cells via modulation of AKT activity by zinc
Source: Metallomics. 2026 Mar 6;18(1):mfag011. doi: 10.1093/mtomcs/mfag011 (PMC13055888; doi:10.1093/mtomcs/mfag011)
Supplement: mfag011_Supplemental_File [file mfag011_supplemental_file.docx]

***Supplementary material for***

SLC30A1, SLC30A5 and SLC30A9 transporters play crucial role in ligand-independent activation of ESR1 signalling in breast cancer cells via modulation of AKT activity by zinc

Szymon Lekki-Porębski^1 2 X^, Michał Rakowski^1^, Agnieszka Grzelak^1^

^1^ Centre for Digital Biology and Biomedical Science - Biobank Lodz, Faculty of Biology and Environmental Protection, University of Lodz, Lodz, 90-236, Poland

^2^ The Bio-Med-Chem Doctoral School of the University of Lodz and Lodz Institutes of the Polish Academy of Sciences, University of Lodz, 90-237 Lodz, Poland

^x^ – corresponding author e-mail: szymon.lekki.porebski@edu.uni.lodz.pl


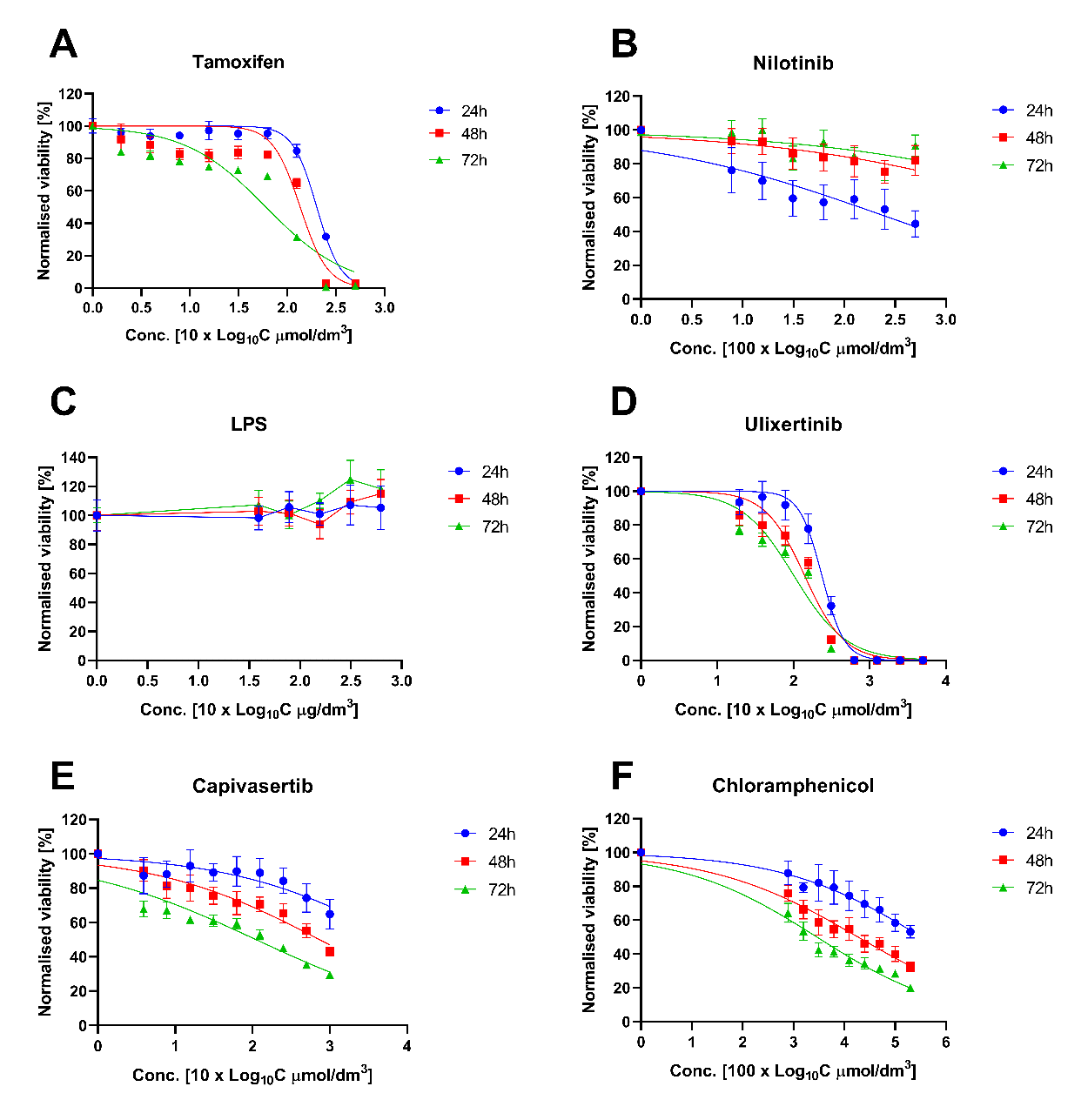


Supplementary Fig. 1: Viability curves of MCF-7 cells incubated with selected factors. The viability of cells was measured using resazurin assay. Viability curves were prepared using a non-linear, five parameter curve model [n = 6].

| Gene name | Gene ID | Sequence | | Primer Lenght | Product Lenght | Source |
| --- | --- | --- | --- | --- | --- | --- |
| *SCL30A1* | 7779 | Forward | 5'-GGACAACTTAACATGCGTGGA-3' | 21 | 128 | Primer Bank |
|  |  | Reverse | 5'-ACACAAAAATCCCCTTCAGAACA-3' | 23 |  |  |
| *SLC30A4* | 7782 | Forward | 5'-TGACCTAAGCGCCATCATACT-3' | 21 | 106 | Primer Bank |
|  |  | Reverse | 5'-AGCTGACAAAACCTCTAAGCG-3' | 21 |  |  |
| *SLC30A5* | 64924 | Forward | 5'-ACCAAACACCAGTGGATCAAAA -3' | 24 | 108 | Primer Bank |
|  |  | Reverse | 5'-CAGCAAAGTCCTTAGTGGTCC -3' | 23 |  |  |
| *SLC30A6* | 55676 | Forward | 5'-AGGAAACCTAGCCCTGTCTATT-3' | 22 | 126 | Primer Bank |
|  |  | Reverse | 5'-CAAAAAGCGTTCTGCACTTTCT-3' | 22 |  |  |
| *SLC30A7* | 148867 | Forward | 5'-TCTCGGGCTGGTTTAGGTCTA-3' | 21 | 88 | Primer Bank |
|  |  | Reverse | 5'-ACAAAAGCGAAAGAGAGGTTCA-3' | 22 |  |  |
| *SLC30A8* | 169026 | Forward | 5'-TGAGTACGCCTATGCCAAGTG-3' | 21 | 144 | Primer Bank |
|  |  | Reverse | 5'-CTGGTCAGGTCAATTAAGAGGTG-3' | 23 |  |  |
| *SLC30A9* | 10463 | Forward | 5'-AGGGAGTATGGCTCAAAGTACA-3' | 22 | 120 | Primer Bank |
|  |  | Reverse | 5'-GGGACTTCGTCGTCTGATTTTTC-3' | 23 |  |  |
| *MT2A* | 4502 | Forward | 5'-GCATCTGCAAAGGGGCGTC-3' | 19 | 127 | Designed |
|  |  | Reverse | 5'-CGGTCACGGTCAGGGTTGT-3' | 19 |  |  |
| *TFF1* | 7031 | Forward | 5'-CCCCGTGAAAGACAGAATTGT-3' | 21 | 81 | Primer Bank |
|  |  | Reverse | 5'-GGTGTCGTCGAAACAGCAG-3' | 19 |  |  |
| *GAPDH* | 2597 | Forward | 5’-GTCTCCTCTGACTTCAACAGCG-3’ | 22 | 131 | Primer Bank |
|  |  | Reverse | 5’-ACCACCCTGTTGCTGTAGCCAA-3’ | 22 |  |  |
| *RPL13A* | 23521 | Forward | 5’-CTCAAGGTGTTTGACGGCATCC-3’ | 22 | 143 | Primer Bank |
|  |  | Reverse | 5’-TACTTCCAGCCAACCTCGTGAG-3’ | 22 |  |  |

Supplementary Fig. 2: Sequence of primers and source of sequences used in RT-qPCR assay.

|  | ***Gene Name*** | |
| --- | --- | --- |
| **Melt curves** | *SLC30A1* | *SLC30A4* |
|  | *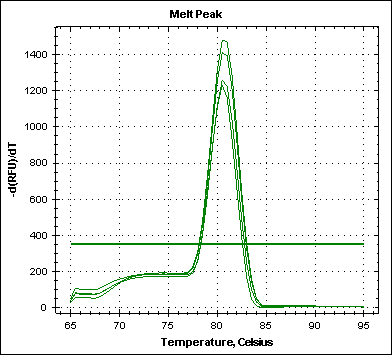* | *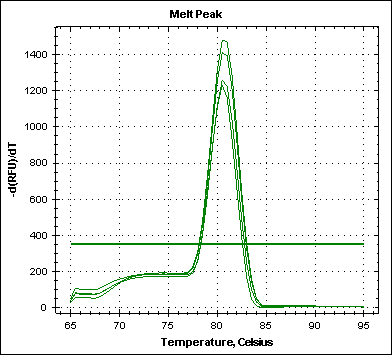* |
|  | *SLC30A5* | *SLC30A6* |
|  | *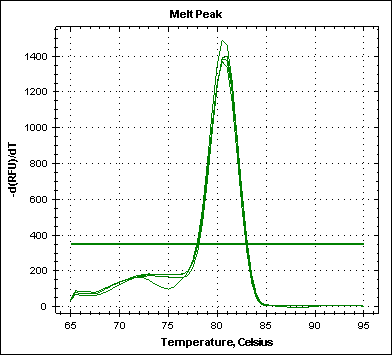* | *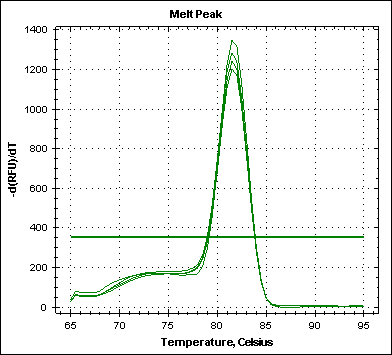* |
|  | *SLC30A7* | *SLC30A9* |
|  | *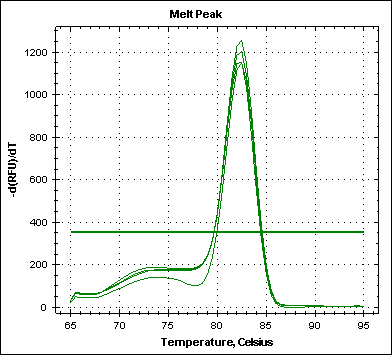* | *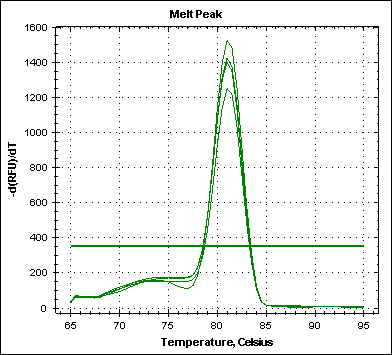* |
|  | *TFF1* | *MT2A* |
|  | *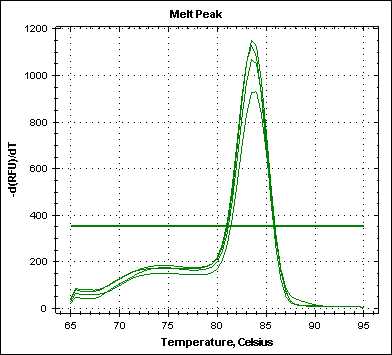* | *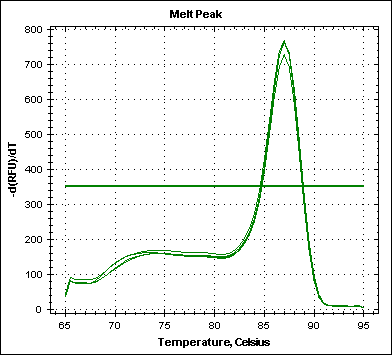* |
|  | *RPL13A* | *GAPDH* |
|  | 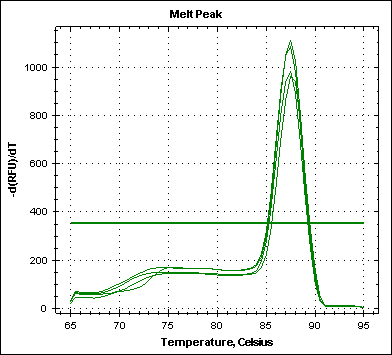 | 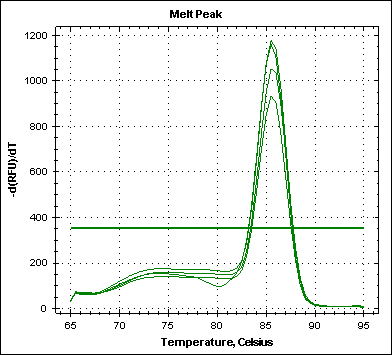 |

Supplementary Fig. 3: Representative melt curves of PCR products


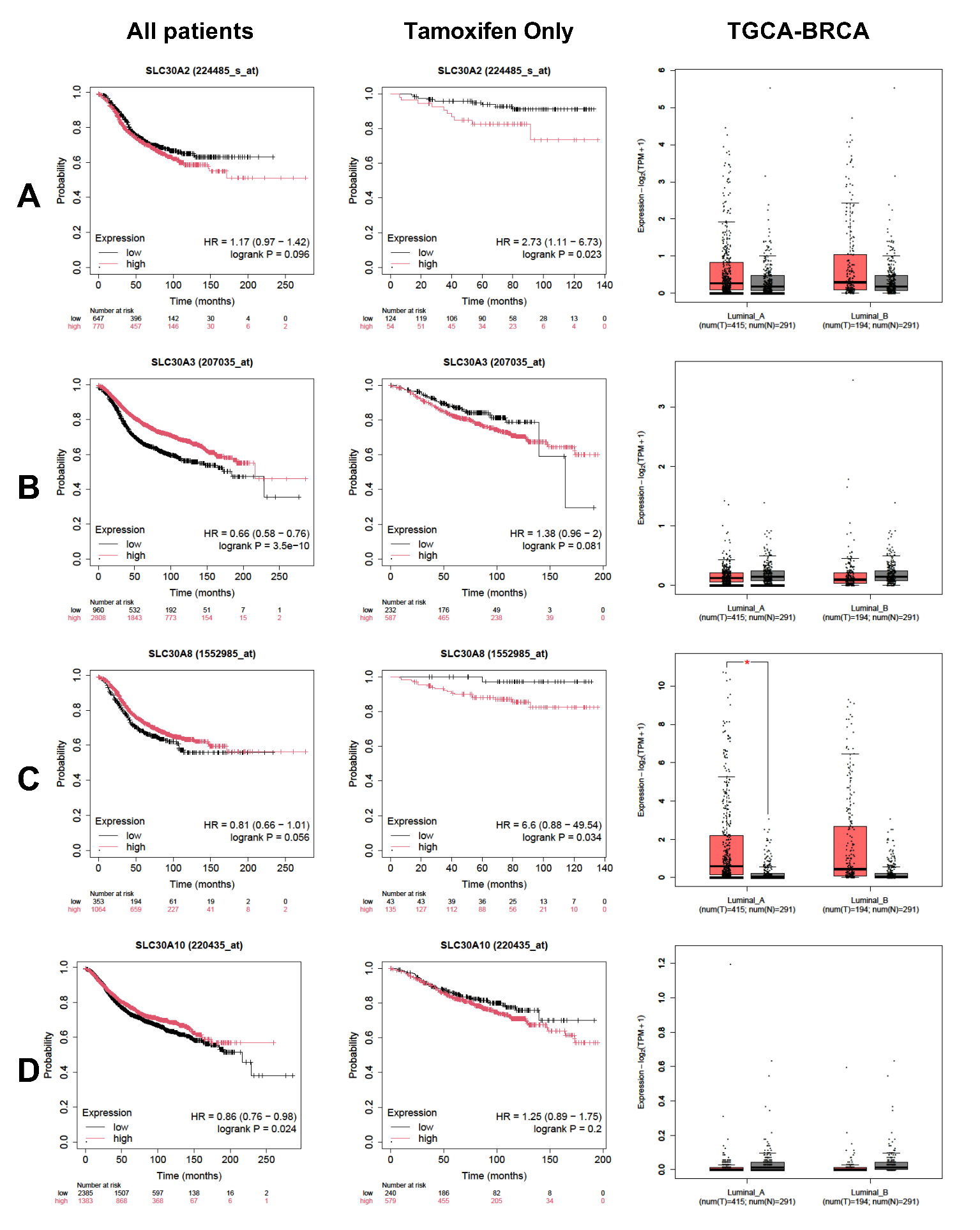


Supplementary Fig. 4: Bioinformatics analysis of *SLC30A2* (A), *SLC30A3* (B), *SLC30A8* (C) and *SLC30A10* (D) genes.

| No. | Pathway symbol | Genes | Publication DOI |
| --- | --- | --- | --- |
| 1 | AKT1 | STAT2 COPG2 TAF15 NRCAM FSTL1 OLR1  WDR19 GRAMD2 PDE5A | 10.1371/journal.pone.0178865 |
| 2 | PRKCD | PPP1R15A SPHK1 TRAF1 SERPINB2 BCL2A1 FOSL1 CCL20 LIPG C3orf52 | 10.1074/jbc.M110.194332 |
| 3 | RAF1 | HSPB1 BCL6 TXNIP CLK1 CRYAB BTG1 IGBP1 GRN HSD11B2 | 10.1091/mbc.e03-11-0807 |

Supplementary Fig. 5: List of genes used as signalling pattern of activation of specific kinase, including reference for studies confirming presence of such patterns in case of those pathways.


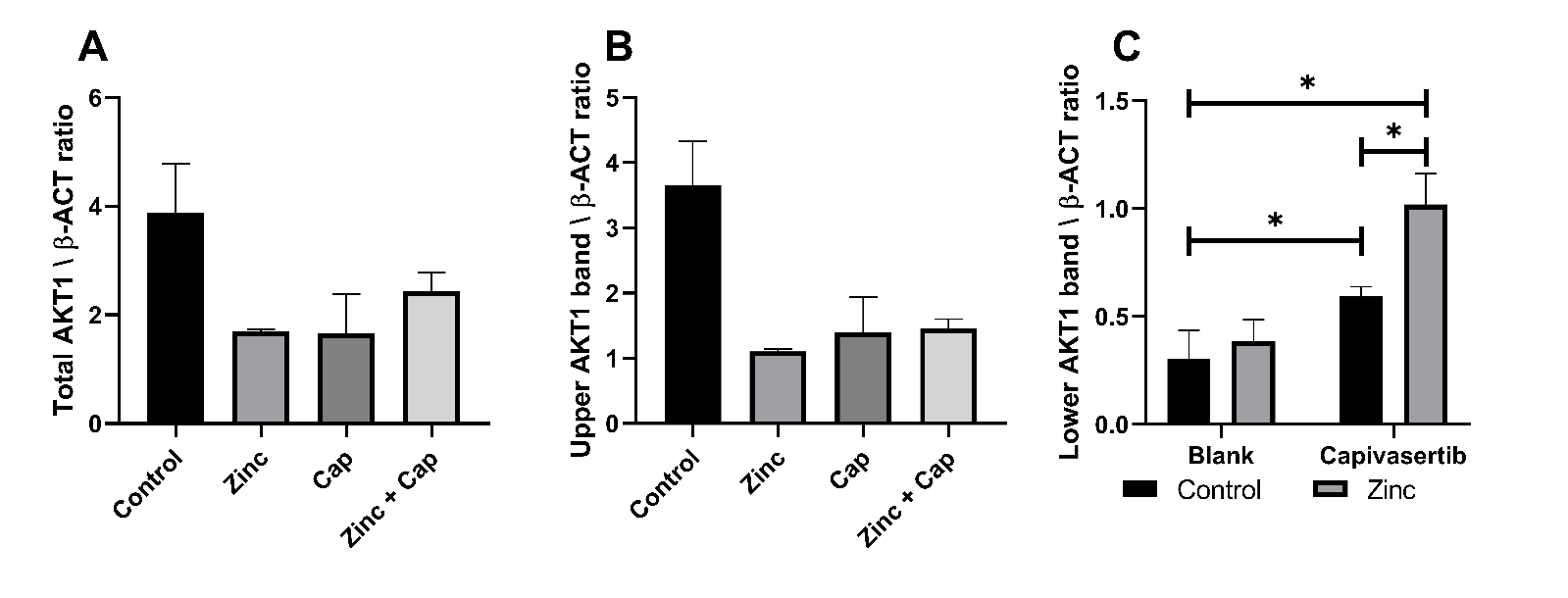


Supplementary Fig. 6: Western Blot value of total AKT bands (lower and upper band – A), probably ubiquitinated AKT1 (Upper band ~56 kDa + 8 kDa, B) and normal AKT1 (Lower band ~56 kDa, C). AKT1 values were normalised using β-actin. Results for A and B were analysed using non-parametric ANOVA and were not statistically significant [n = 3]. Results for C were analysed using parametric ANOVA [n = 3]. * - p <0.05

| No | β-ACT | p-AKT1 & AKT1 | Light |
| --- | --- | --- | --- |
| 1 |  |  | 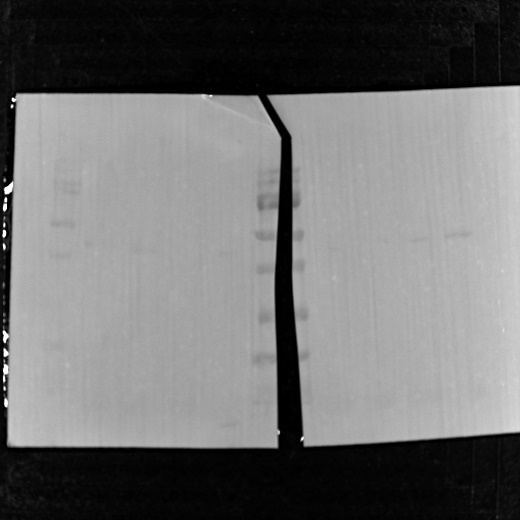 |
| 2 |  |  | 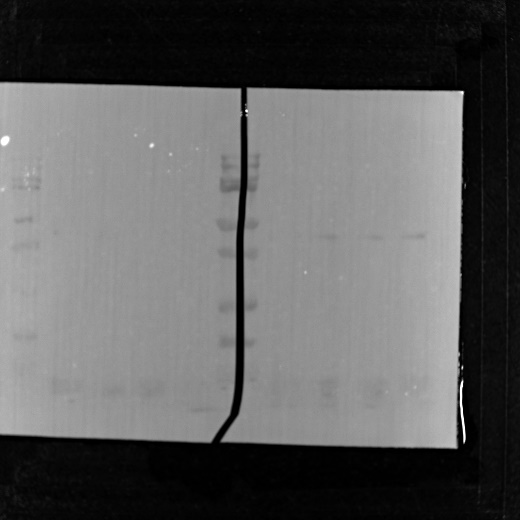 |
| 3 |  |  | 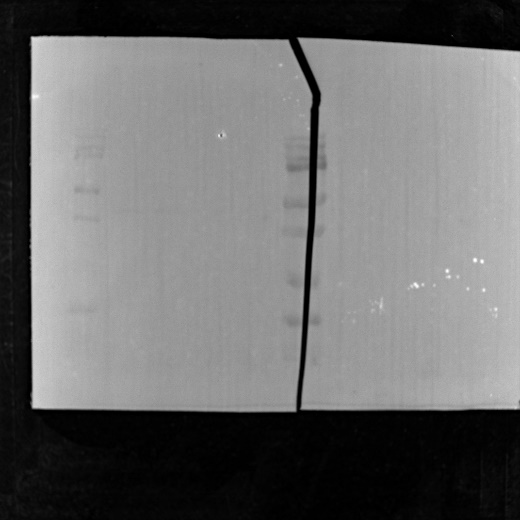 |

Supplementary Fig. 7: Western Blot photographs of chemiluminescence value of either β-ACT or AKT1 and light photographs of membranes. Lanes 1 & 5 contains lane weight marker, 2 & 7 - control samples, 3 & 8 zinc incubated samples, 4 & 9 capivasertib incubated samples, 5 & 10 capivasertib + zinc incubated samples. Left half of membrane (lanes 1-4) was incubated with Anti-phospho-AKT1 antibodies and right half (lanes 6-10) with Anti-AKT1 antibodies. Lanes from membrane No 3 were used in manuscript in figure 3.


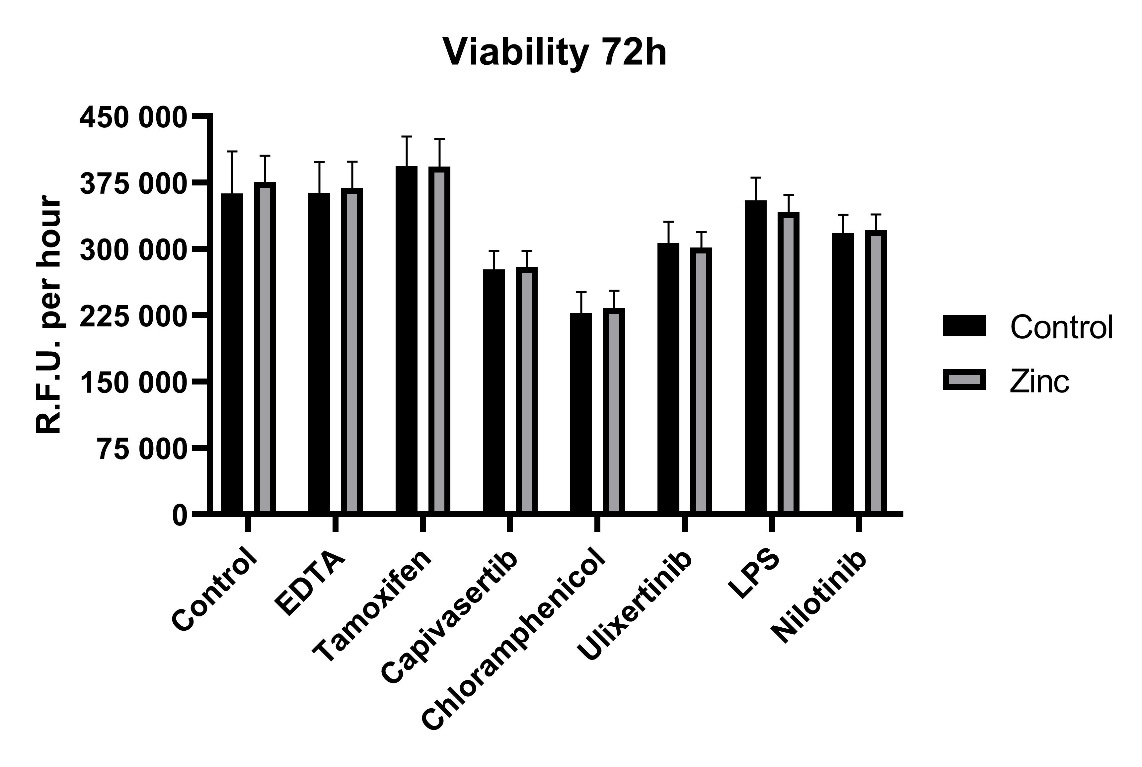


Supplementary Fig. 8: Unnormalised viability of cells incubated 72 hours with either zinc donor and/or specific inhibitor. Results were analysed using Results were analysed using paired multiple T-test [n = 12]. Analysis was performed comparing zinc donor and zinc free variants. Results did not exhibit any statistical significance between each analysed pair (minimal p > 0.17).
